# Evaluating annotations of an Agilent expression chip suggests that many features cannot be interpreted

**DOI:** 10.1186/1471-2164-10-566

**Published:** 2009-11-30

**Authors:** E Michael Gertz, Kundan Sengupta, Michael J Difilippantonio, Thomas Ried, Alejandro A Schäffer

**Affiliations:** 1 Computational Biology Branch, National Center for Biotechnology Information, National Library of Medicine, National Institutes of Health, DHHS, Bethesda, MD-20892, USA; 2 Section of Cancer Genomics, Genetics Branch, Center for Cancer Research, National Cancer Institute, National Institutes of Health, DHHS, Bethesda, MD-20892, USA

## Abstract

**Background:**

While attempting to reanalyze published data from Agilent 4 × 44 human expression chips, we found that some of the 60-mer olignucleotide features could not be interpreted as representing single human genes. For example, some of the oligonucleotides align with the transcripts of more than one gene. We decided to check the annotations for all autosomes and the X chromosome systematically using bioinformatics methods.

**Results:**

Out of 42683 reporters, we found that 25505 (60%) passed all our tests and are considered "fully valid". 9964 (23%) reporters did not have a meaningful identifier, mapped to the wrong chromosome, or did not pass basic alignment tests preventing us from correlating the expression values of these reporters with a unique annotated human gene. The remaining 7214 (17%) reporters could be associated with either a unique gene or a unique intergenic location, but could not be mapped to a transcript in RefSeq. The 7214 reporters are further partitioned into three different levels of validity.

**Conclusion:**

Expression array studies should evaluate the annotations of reporters and remove those reporters that have suspect annotations. This evaluation can be done systematically and semi-automatically, but one must recognize that data sources are frequently updated leading to slightly changing validation results over time.

## Background

Agilent-014850 Whole Human Genome Microarray 4 × 44K G4112F consists of 43,376 oligonucleotides 60 nucleotides in length, most of which are annotated as corresponding to the sequence of a known or predicted human gene, along with a number of probes that function as controls. Agilent supplies an annotation file for the array. The file provides the sequence of each oligonucleotide, its position on the NCBI human reference assembly, and the gene and transcript putatively associated with it. The annotation file states "This multi-pack (4 × 44K) formatted microarray represents a compiled view of the human genome as it is understood today." From here on, we refer to each oligonucleotide as a "reporter", rather than the more commonly used terms oligo, probe, or 60-mer, to indicate that each oligonucleotide is supposed to "report" the expression of a single gene unambiguously. Some reporters unambiguously distinguish between transcripts of a single gene, but others hybridize with more than one transcript of the same gene.

We sought to validate the annotations against the current information for the human genome. Preliminary analysis showed that a substantial number of reporter annotations are questionable, and that other reporters may not be a specific representation of a unique gene. Moreover, some reporter annotations are incomplete and fail to provide a discernible link between an oligonucleotide and a human RNA. We therefore validated the reporter annotations against the data present in the NCBI Entrez [1] database http://www.ncbi.nlm.nih.gov. Because numerous reporters were annotated with an identifier from the Gene Index Project but not recognized by Entrez, we used the Gene Index Project [2] as an additional source of transcript data.

Entrez is a collection of related databases of biological data, among which are the Entrez Nucleotide database of biological sequences, the RefSeq [3] database providing reference chromosome assemblies and transcribed sequences for several species, and the Entrez Gene [4] database cataloging known genes. Agilent includes the RefSeq RNA identifier and Entrez Gene identifier as standard fields in the annotation file. The RefSeq identifier is, however, supplied for only approximately two thirds of the oligonucleotides on the chip.

The Entrez databases are frequently updated. As our knowledge of the human genome increases, RNA and gene records are added and edited. Unreliable records are deleted or suppressed. Major changes to the data of a gene merit a change in the identifier of the RefSeq RNAs, or even of the Entrez Gene record. We therefore sought to associate the oligonucleotides on the array with human genes using the BLAST [5-7] and Splign [8] alignment algorithms, and cross references between current database records, rather than relying on the given identifiers. Since Entrez databases have been updated multiple times after the Agilent annotation file was prepared, some discrepancies were expected. In concept, this study is similar to a study performed by Gaj et al. [9] who evaluated the annotations against protein databases, which do not include the increasing number of non-protein coding genes. The method and programs (which are freely available for download; see Methods) used to perform this validation should be applicable to other gene chips.

## Results

We partitioned the reporters into five distinct categories of descending confidence: fully valid; Refseq RNA valid; other gene valid; possibly valid; and invalid. Lists of these reporters, and where possible, the associated genes and transcripts, are provided as additional files 1, 2, 3, 4 and 5. Counts of the number of reporters in each category are shown in Table 1; for all tables, our results were tabulated by chromosomes, but since we detected no chromosome-specific effects, only genome-wide totals are shown. *Fully valid *reporters can be associated with the transcripts of a single gene and with chromosomal locations that lie within the same gene. *Refseq RNA valid *reporters are associated with the RefSeq RNA transcripts of a single gene, but for one of the reasons described below cannot be placed within the chromosomal extent of a single gene. *Other gene valid *reporters can be placed on the chromosome within the location of a single gene, but the reporter either lies in an intron of that gene or in a transcript not yet in RefSeq. *Possibly valid *reporters can be placed at a unique location on the human reference assembly, but this location is not the position of a gene currently in Entrez Gene. Despite the lack of solid evidence to associate a possibly valid reporter to a gene, for studies relating expression levels to genomic positions (e.g., reference [10]), it makes sense to include these reporters. The division into categories is dependent on the current contents of the RefSeq and Entrez database, which are frequently changing.

**Table 1:** Reporters divided into the five categories discussed in Results

Fully Valid	RefSeq RNA Valid	Other Gene Valid	Possibly Valid	Invalid	Total
25505	1859	2187	3168	9964	42683

The far right column shows the total number of eligible reporters, and the other entries show the total number of reporters in each category.

As explained in Methods, we restricted our analysis to the 42683 reporters labeled as being on an autosome or on the X chromosome. We consider 25505 (60%) reporters to be fully valid and associated with a single gene in RefSeq. A fully valid reporter must have MegaBLAST alignments to the RefSeq RNA transcripts of exactly one gene in Entrez Gene, and at least one of those alignments must be high-quality as defined in Methods. An example of a reporter that fails the uniqueness test is 1591^a ^because it has 100% identical matches to two related genes *PRAME16 *and *PRAME17*. The alignment to a transcript should be in the forward ("sense") direction; reporters that have only alignments in the reverse direction are invalid. If a reporter has alignments in the reverse direction, but also has at least one high-quality forward alignments to a RefSeq RNA whose status is more definite than "model", then the reverse alignments are ignored and the reporter remains under consideration for being fully valid or RefSeq RNA valid. Table 2 summarizes the results of aligning reporters to RefSeq RNA. The distinction between fully valid and Refseq RNA valid is that fully valid reporters are placed by Splign on the reference assembly of chromosome specified by Agilent and within the Entrez extent of exactly one gene, which must be the same gene found by alignment to RefSeq RNA. Fully valid reporters are permitted to have additional alignments to their corresponding gene, to the reverse complement of a different gene, to intergenic regions, or to untranscribed pseudogenes.

**Table 2:** Results of aligning all eligible reporters to the database of human RefSeq RNAs

Eligible	Rev. Comp.	Multiple Genes	Unique Gene	No Alignment
42683	2009	2463	27364	10847

The first column show the total number of eligible reporters. The second column shows the number of reporters eliminated because they align to the reverse complement of a RefSeq RNA. The third column shows the number of reporters eliminated because they align to the transcripts of more than one gene. The fourth column lists the number of reporters that align to the transcripts of a single gene. The fifth column shows the number of reporters that did not have a sufficiently high-scoring alignment to a RefSeq RNA. Reporters that could be counted in more than one column of the third through fifth columns are counted in the leftmost column.

There are 1859 (4%) reporters that align to the transcripts of a single RefSeq gene, but that Splign did not place within the accepted extent of exactly one gene. We do not consider such reporters to be fully valid, but rather place them in the category of "Refseq RNA valid." Reporters in this category fall into four distinct classes (Table 3). The first class consists of reporters not placed within the accepted extent of any gene. For example, ID 22872^b ^is supposed to match gene *SLC35D1*, but maps approximately 3.6 kbp away from the RefSeq extent of *SLC35D1*. The second class includes reporters placed within the extent of more than one gene. The additional alignments may be to introns, or may instead be parts of transcripts not yet included in the RefSeq RNA database. The third class includes reporters that are placed at a single location on the reference assembly, but this position lies within the extent of more than one gene because the genes themselves overlap. For example, reporter 5099^c ^matches gene *KTI12*, which lies in an intron of another gene *TXNDC12*. We consider it likely that reporters in this third class identify the transcripts of a single gene, but to avoid potential confusion we do not include these reporters in the list of those that are fully valid. The fourth class consists of reporters that align to a different gene than the one found by aligning to RNA transcripts. We did not need to consider the class of genes on chromosome X that are duplicated on the Y chromosome; no reporters mapped to the positions of these genes.

**Table 3:** Results of placing the reporters that align with the RefSeq RNA transcripts of a single gene on the chromosome

Eligible	Fully Valid	No Placement	Multiple Genes	Overlapping Genes	Wrong Gene
27364	25505	177	1429	238	15

The first column is the total number of eligible reporters, which is the same data as the fourth column of Table 2. The second column shows the number of reporters that have a valid placement in a unique gene; this is the same data as the first column of Table 1. The third column shows the number of reporters that did not have a high-quality placement. The fourth column shows the number of reporters that have multiple placements and are placed in the location of more than one gene. The fifth column shows the number of reporters with a single placement, but a placement within overlapping genes. The sixth column shows the number of reporters placed in a single gene, but not the same gene as found by alignment to RNA. The sum of the four rightmost columns gives the 2565 RNA valid reporters in Table 1.

We do not require that the GeneID associated with a reporter during the validation process matches the GeneID supplied by Agilent, nor that the chromosomal positions match exactly. GeneIDs may differ because the Entrez and RefSeq databases are not static, and sometimes updates to records merit changes in an identifier. The positions found by Splign sometimes disagree with the positions supplied by Agilent because a splice site occurs within the alignment, and Splign resolves the splice differently. There are also 87 reporters that are fully valid, but align to more than one position within their identified gene.

Excluding the 27364 (64%) fully valid and Refseq RNA valid reporters leaves 15319 (36%) reporters. Among those, 4472 were found to be invalid by the tests of alignment to Refseq RNA (Table 2: sum reverse complement + multiple genes), leaving 10847 reporters for further evaluation. We were able to associate 7603 of these with a putative transcript included in Entrez Nucleotide or the Gene Index Database [1] using the identifier provided in the annotation file; the remaining 3244 (10847 - 7603) reporters were considered invalid and were excluded from further consideration by rules described in Methods (see Table 4).

**Table 4:** Counts of reporters associated with a putative transcript not in RefSeq

Eligible	NoID	Suppressed	No Alignment	Valid ID
10847	2114	845	285	7603

The first column shows the number of reporters that did not have an alignment with a RefSeq RNA; these data are also shown as the fifth column of Table 2. The second column shows the number of reporters that had no discernible identifier in the annotation file. The third column shows the number of reporters for which the annotated identifier had been suppressed or removed in Entrez Nucleotide. The fourth column shows the number of reporters that did not align with the annotated sequence. The fifth column shows the number of reporters that could be associated with a putative transcript. Reporters that could be counted in more than one of the second through fourth columns are counted in the leftmost column.

We used Splign to place the remaining 7603 reporters on the human reference assembly. Table 5 summarizes the results of performing this placement. Of the 7603 reporters, 2187 are placed within the extent of exactly one gene, and this gene is on the chromosome specified in the annotation file. We refer to these reporters as "other gene valid". Reporters that also align to the reverse complement of a gene are not included in this category, because we do not have sufficient evidence to suggest that the forward copy, rather than the reverse, is transcribed. Moreover, 3168 of the 7608 reporters may be placed at a single position on the reference assembly, though this position is not the location of a known gene. We categorize these reporters as "possibly valid" (Column 5, table: 1). Any secondary placement of a reporter suffices to exclude it from this category, because Entrez Gene does not provide sufficient evidence to determine which unit of transcription, if any, the reporter measures.

**Table 5:** Results of placing the reporters that do not align with a RefSeq RNA transcript

Eligible	No Placement	Multiple Genes	Overlapping Genes	Wrong Chromosome	Other Gene Valid	Possibly Valid
7603	1662	389	91	83	2187	3168

The first column is the total number of eligible reporters, which is the same data as the fifth column of Table 4. The second column shows the number of reporters that could not be placed. The third column shows the number of reporters that have multiple placements and are placed in the location of more than one gene. The fourth column shows the number of reporters with a single placement, but a placement within overlapping genes. The fifth column shows the number of reporters with a unique placement on an unexpected chromosome. The sixth column shows the number of reporters that could be associated with a unique gene and thus are considered positionally valid. The seventh column shows the number of reporters that could be associated with a unique placement, not on a gene. The sixth and seventh columns of this table are the same as the third and fourth columns of Table 1. Reporters that could be counted in more than one column of the second through fifth columns are counted in the leftmost column.

## Discussion

We initiated a study to re-evaluate published expression data that were suspicious because they suggested that some mouse genes hybridized better to the Agilent 4 × 44 chip than the orthologous human gene, even though the chip is designed to hybridize to human genes [10]. Preliminary sequence analysis showed that some of the reporters did not align to any known human RNA, and others aligned only to reverse complements. Therefore, we decided to check the annotation file reporter by reporter. To our surprise, approximately 23% of the reporters failed basic tests and could not be assigned any meaning. Thus, the signal intensities of these reporters, which we call "invalid", do not provide useful information as to the expression level of a specific gene. While we had expected some reporters to be problematic - perhaps 5-10% - due to updates to information about the human genome, we did not, however, expect that as much as 40% of the reporters on a 44K gene expression array would for one reason or another yield uninterpretable data. The distinction between 40% uninterpretable and the earlier 23% includes reporters that map uniquely to the human genome but not to a region covered by a RefSeq transcript; for example, this includes reporters that map to an intron.

A search with PubMed and Google found that numerous studies have used Agilent 4 × 44K human expression chips; we cite 10 examples from different research groups [11-20]. Each paper has a small subsection in Methods explaining how the 4 × 44 arrays were hybridized and how the data were analyzed. None of these 10 studies seems to have considered the possibility that the annotations provided do not correspond unambiguously to human genes. The Methods in two studies [12,13] describe some steps to restrict analysis to those reporters for which the Agilent annotation includes a human gene symbol. Another study [19] used a pre-established list of genes, but does not explain how gene identifiers were matched to the annotation file. The lack of a gene symbol in the annotation file eliminates 6089 of the 9964 reporters we consider invalid, and also eliminates 799 of the 25505 fully valid reporters, 148 of the 1859 Refseq RNA valid reporters, 1273 of the 2187 other gene valid reporters, and 2470 of 3168 possibly valid reporters. Of the 31904 reporters with a gene symbol, we consider 24706 (77%) fully valid. Our validation of the annotated transcript identifier invalidates fewer reporters than a test for the presence of a gene symbol, and would not, by itself, be an effective filter. An example of a fully valid reporter that is not annotated with a gene symbol or the identifier of a transcript is reporter ID 27386, which aligns perfectly to transcripts of the gene *RPP14 *(GeneID:11102).

Standards for microarray experiments, such as MIAME [21], are evolving. Generally, these standards seek to ensure that the experiments and data analysis are sufficiently detailed so as to be reproduced at another site. The MIAME paper [21] does recognize the potential for annotation problems, stating, for example, that "Because references to an external gene index may not be stable, it is essential to physically identify each element's composition. Disclosing the nature of the relationship between an array element and its cognate gene's transcript allows informed assessment..." However, there seems to be no easy-to-enforce mechanism to ensure that the annotations that come with an expression array are internally consistent, let alone consistent with changing external genomic databases. So long as researches follow MIAME and deposit data in detail, it is possible to apply reporter validation post-facto.

## Conclusion

We hope that our reporter-by-reporter validation of the 4 × 44K array annotations will prove useful for labs using these chips. Our recommendations are the following:

1. It is safe to use the 25505 fully valid reporters.

2. It is unsafe to use the 9964 invalid reporters.

3. The 4046 reporters that are either Refseq RNA valid or other gene valid might be used for gene-based studies.

4. The 7214 reporters that are in any of the three intermediate categories (Refseq RNA valid, other gene valid, possibly valid) might be used for position-based studies.

5. In any study that uses reporters in the three intermediate categories and reports either genome-wide or chromosome-wide results, the analysis should be repeated with the fully valid reporters only to verify that this more reliable subset provides qualitatively identical results.

## Methods

The software we developed for this study is available at ftp://ftp.ncbi.nlm.nih.gov/pub/microarray_pipeline. Because some of the data sources change over time, readers may wish to rerun the analysis. Alternatively, readers may wish to modify the software to analyze other microarray annotation files.

### Databases

We validated reporter annotations using the data current in Entrez on September 29, 2009. On this date, the current reference assembly was build 37.1, and the version number of RefSeq was 37. Each transcript in RefSeq is linked with an Entrez Gene database identifier (GeneID). Thus, given a RefSeq transcript, it is possible to find its GeneID. Conversely, for any human GeneID, one may determine the list of all transcripts for that gene that appear in RefSeq. The Entrez Gene database is the direct successor of the LOCUSLINK database, which is mentioned in the Agilent annotation file. The two databases use the same identifiers.

We retrieved transcript data from the Gene Index Database on May 29, 2009. On this date, the release number for the human genome was 17.0. We performed a batch retrieval using the web interface http://compbio.dfci.harvard.edu/tgi.

### Microarray Annotation File

We analyzed the oligonucleotides on the Agilent-014850 Whole Human Genome Microarray 4 × 44K G4112F. Agilent provides an annotation file, putatively associating reporters with expressed human RNAs, and these RNAs with their respective genes. Agilent sometimes releases updated versions of the annotation file. We used a version from late 2007 that was used in a previous study [10]. The latest version of the annotation file, released April 16, 2009, gives nearly identical results for fully valid and RefSeq RNA valid reporters; the only differences arise for a few reporters that have been assigned a different chromosome in the newer annotation file. Using the newest annotation file does not qualitatively change the results for the other gene valid, possibly valid, or invalid markers.

Of the 43,376 non-control oligonucleotides on the array, 414 had no chromosome labeled, 20 were associated with the mitochondrial genome, 133 were associated with the Y chromosome, and 126 were labeled with the qualifier "random". We did not consider any of these reporters, choosing instead to focus on the 42,683 reporters associated with the autosomes and the X chromosome.

### Aligning Nucleotide Sequences

Except where otherwise stated, we aligned nucleotide sequences using NCBI's MegaBLAST program, version 2.2.21, with default settings. MegaBLAST is optimized for nearly identical sequences. By default, MegaBLAST filters the query sequence to exclude low-complexity regions. Thus, it may find no alignments for some reporters that contain repetitive DNA. The use of this option is appropriate, as we have low confidence that repetitive DNA may be used to identify a unique gene.

MegaBLAST has an option "-S" that causes it to search a database of nucleotide sequences using the query only in the forward sense, or only in the reverse complement sense. We use this option as appropriate without further comment.

### Aligning Reporters with RefSeq Human Transcripts

We define that an oligonucleotide *aligns *with a human RNA transcript if MegaBLAST [8] with default parameters reports such an alignment. By default, MegaBLAST finds alignments between two sequences only if, after the query has been filtered to mask low-complexity sequences, the sequences still have a gapless alignment of length at least 28, sometimes relaxed to length 16. The alignment ultimately reported by MegaBLAST may be a higher-scoring alignment with gaps.

**Figure 1:**
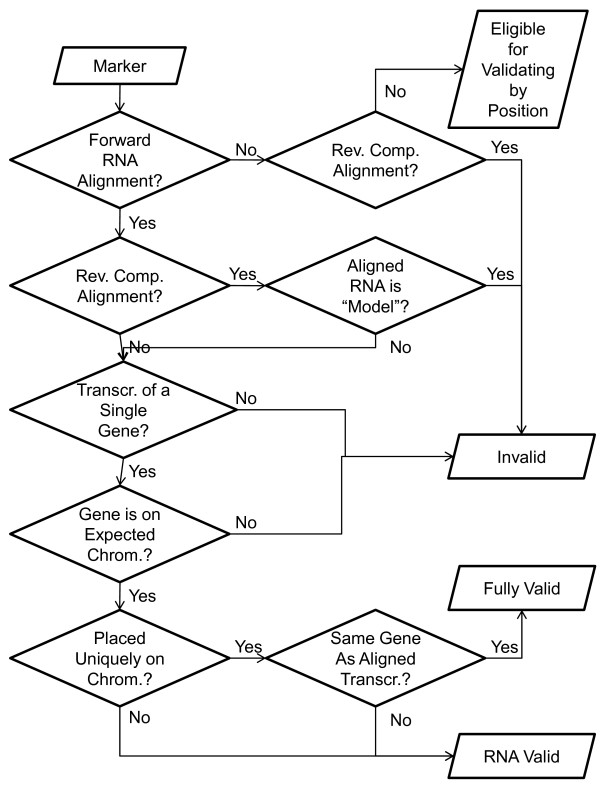
**Flowchart showing the process, described in Methods, used to include a reporter in one of the top two categories, invalidated, or declare it eligible for validation for position**. The process for validating by position is shown in Figure 2.

To confirm the association of a reporter with a gene, we require that the alignment have a score of at least 100 bits; we refer to such alignments as *high quality *alignments. With the command-line MegaBLAST's default scoring system, a perfect alignment has a score of 119 bits; the web-enabled version uses a different default scoring system. Each mismatch in a full length alignment reduces the score by approximately eight bits, whereas each mismatch at the end of an oligonucleotide reduces the score by approximately two bits. Figure 1 shows a flowchart of the process used to validate a reporter through alignment to RefSeq RNAs.

### Comparing Reporters to their Annotated Sequence

We collected the set of reporters that were not validated and eliminated by alignment to a RefSeq human gene transcript. For each of these reporters, we searched field 15 of the annotation file for a sequence identifier. We preferred, in order, a RefSeq identifier, a GenBank [22] accession number, or an identifier from the Gene Index Project. GenBank accession numbers can be used to retrieve records from Entrez Nucleotide. The type of identifier was determined by the format. RefSeq identifiers start with the string "ref"; GenBank identifiers start with "gb"; and identifiers from the Gene Index Project start with "thc". We retrieved the sequences putatively associated with each reporter by the annotation file from either Entrez Nucleotide or from the Gene Index Project. At this stage, we eliminated reporters for which no identifiable transcript identifier could be found in the annotation file, or for which the identified sequence could not be found in either database. If the sequence was annotated within Entrez Nucleotide as replaced or suppressed, we excluded the corresponding reporter from further consideration.

We used MegaBLAST to align each candidate reporter with its annotated sequence. MegaBLAST's parameters were relaxed to use a word size of 16 and to omit low-complexity filtering, but an alignment was still required to have a score of at least 100 to be considered a significant match. Those reporters that had no significant match to their annotated sequence were eliminated from further consideration. For example, reporter 5467^d ^is supposed to match NM_182578; the oligo does align to an obsolete version NM_182578.1, but not to the current version NM_182578.2. Therefore, 5467 was excluded.

### Validating Reporters by Placement on the Reference Assembly

**Figure 2:**
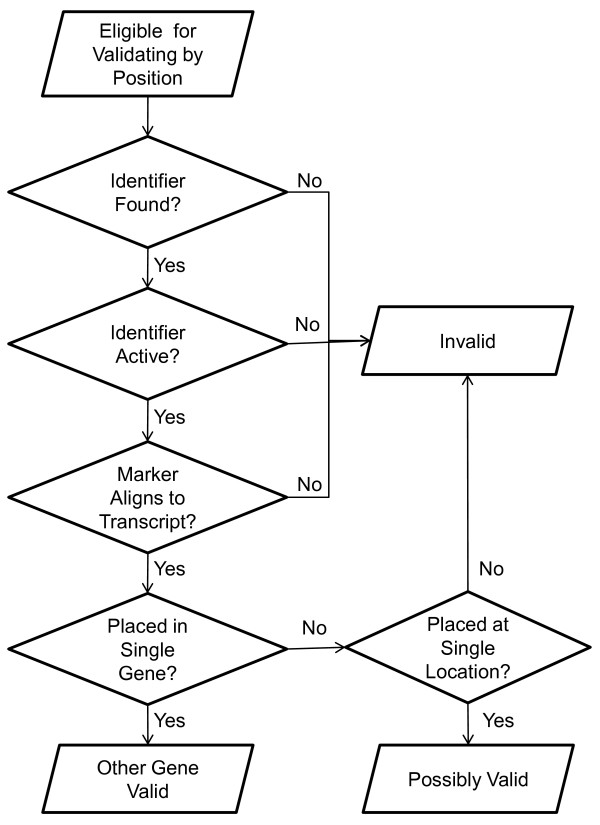
**Flowchart showing the process, described in Methods, used to validate a reporter by position, placing it into one of the categories: other gene valid, possibly valid, or invalid**.

We used Splign to place candidate reporters on the assembled chromosomes of the human reference assembly. We aligned reporters to chromosomes using MegaBLAST with word size 16, but otherwise used default parameters. We used the resulting alignments as input to Splign, which takes splice sites into consideration when placing reporters. We say a reporter is placed at a location on a chromosome if this combination of MegaBLAST with Splign reports such a placement, and at least 40 of 60 bases in the oligonucleotide match the chromosomal sequence. A placement that matches at least 50 of the 60 is considered a high-quality alignment. For each placement on a chromosome, we found the entry in Entrez Gene, if any, that intersects with that position. If the gene was assigned a position in MapViewer [2], then we used that position. Otherwise, we took the union of all the RefSeq transcripts for the gene to determine the widest possible extent. Figures 1 and 2 show flowcharts of the process used to validate reporters by placement on the reference assembly.

## Authors' contributions

EMG designed the content and sequence of tests in the two Figures, carried out the data analysis and wrote most of the paper. KS, MJD, TR, and AAS conceived the study. AAS did some pilot analyses hinting at some of the problems discovered by EMG. AAS wrote the Discussion. All authors edited the paper. All authors have read and approved the final version.

## Appendix - Oligonucleotide Sequences

^a^CCTCAAGAACCCCTTGGGAACCTTTATATTCTGTCATGCTTACCTAGCTGATCAGGACAT

^b^AACAGGACTCTGTGTCTCATTGCCTCTAGAATCCTTAGGTAGCAGTTTCTGTTCACTGTC

^c^GAATTGTTTTAAAACAATTGTGAACAGAAACTGAAGATGGTACAGTTCTACATCTGCACC

^d^TATCACGCTCCTCCCACACCCACCCTGGCTTCCATCATGAGTGCAGTGTCATCTCAGCGG

## Supplementary Material

Additional file 1 **List of fully valid reporters, supplied as a tab delimited text file with CR LF line endings**. The columns of the file represent 1) the identifier supplied in the first column of the Agilent annotation file; 2) the Entrez Gene identifier of the corresponding gene; 3) the chromosome matched by the reporter; 4) the start position of the placement of the reporter on build 36 of the NCBI reference assembly; 5) the end position of the placement of the reporter; 6) the orientation of the placement of the reporter; and 7) a semicolon delimited list of RefSeq transcripts matched by the reporter. Some reporters are placed at more than one location within the gene; for these, the start and end positions are marked with an asterisk. Click here for file

Additional file 2 **List of Refseq RNA valid reporters**. The format is similar to the format of additional file 1, except chromosomal positions are not supplied. Click here for file

Additional file 3 **List of reporters classified as "other gene valid."** The format is similar to the format of additional file 1, except transcript identifiers are not provided. Click here for file

Additional file 4 List of reporters classified as "possibly valid." The format is similar to the format of additional file 1, except gene and transcript identifiers are not provided. Click here for file

Additional file 5 **List of invalid reporters**. Click here for file
